# Lithium use in childhood and adolescence, peripartum, and old age: an umbrella review

**DOI:** 10.1186/s40345-023-00287-7

**Published:** 2023-02-12

**Authors:** Delfina Janiri, Gaia Sampogna, Umberto Albert, Filippo Caraci, Giovanni Martinotti, Gianluca Serafini, Alfonso Tortorella, Alessandro Zuddas, Andrea Fiorillo, Gabriele Sani

**Affiliations:** 1 Department of Neuroscience, Section of Psychiatry, Università Cattolica del Sacro Cuore, Largo Francesco Vito 1, 00168 Rome, Italy; 2 Department of Psychiatry, Fondazione Policlinico Universitario Agostino Gemelli IRCCS, Rome, Italy; 3 Department of Psychiatry, University of Campania “L. Vanvitelli”, Naples, Italy; 4 Department of Medicine, Surgery and Health Sciences, University of Trieste, Trieste, Italy; 5 Department of Mental Health, Azienda Sanitaria Universitaria Giuliano Isontina - ASUGI, Trieste, Italy; 6 Department of Drug and Health Sciences, University of Catania, 95125 Catania, Italy; 7 Oasi Research Institute-IRCCS, 94018 Troina, Italy; 8 Department of Neurosciences, Imaging and Clinical Sciences, Università degli Studi G. D’Annunzio, 66100 Chieti, Italy; 9 Department of Neuroscience, Rehabilitation, Ophthalmology, Genetics, Maternal and Child Health (DINOGMI), Section of Psychiatry, University of Genoa, Genoa, Italy; 10 IRCCS Ospedale Policlinico San Martino, Genoa, Italy; 11 Department of Psychiatry, University of Perugia, Perugia, Italy; 12 Department of Biomedical Sciences, Section of Neuroscience & Clinical Pharmacology, University of Cagliari, Cagliari, Italy; 13 Child & Adolescent Neuropsychiatry Unit, “A. Cao” Paediatric Hospital, Cagliari, Italy

**Keywords:** Lithium, Childhood, Adolescence, Paediatric, Peripartum, Postpartum, Pregnancy, Lactation, Elderly, Old age

## Abstract

**Background:**

Lithium is one of the most consistently effective treatment for mood disorders. However, patients may show a high level of heterogeneity in treatment response across the lifespan. In particular, the benefits of lithium use may vary in special clinical conditions. The aim of this study was to test this hypothesis by conducting an umbrella review on the efficacy and safety of lithium in childhood and adolescence, peripartum and old age.

**Methods:**

We applied the Preferred Reporting Items for Systematic Reviews and Meta-analyses criteria (PRISMA) to identify systematic reviews/meta-analyses on the efficacy and/or safety of lithium in mood disorders in special clinical conditions: (i) childhood and adolescence; (ii) peripartum (pregnancy, postpartum and lactation); (iii) old age. The Risk of Bias Assessment Tool for Systematic Reviews (ROBIS) tool was used to assess the risk of bias. Overlap in primary studies across systematic reviews was calculated through the Corrected Covered Area (CCA).

**Results:**

We included 20 independent studies, for a total of 8209 individuals treated with lithium. Regarding paediatric age, efficacy and safety results suggested that lithium may be superior to placebo in bipolar disorders (BD) and not associated with serious adverse events. Nevertheless, primary available data are very limited. Efficacy in paediatric major depressive disorder (MDD) is not clear. During peripartum, lithium use was superior to non-lithium in preventing mood episodes and it was associated with low risk of congenital anomalies and with normal child neurodevelopment. Regarding old age, limited evidence supported lithium as an effective treatment in BD and resistant MDD; low doses should be used in this population. Systematic reviews on paediatric age showed the lowest risk of bias (80% of the studies at low risk). The CCA range of included studies was 13–47%.

**Conclusions:**

This umbrella review supports the use of lithium across the lifespan, including special clinical condition. Nevertheless, more studies with increased methodological homogeneity are needed.

**Supplementary Information:**

The online version contains supplementary material available at 10.1186/s40345-023-00287-7.

## Introduction

Lithium is one of the most consistently effective drug treatment for mood disorders (Kessing et al. 2018; Geddes et al. 2004; Baldessarini et al. 2019). It has been approved for both adults and children and it is currently used from the age of 12 years until old age. However, clinical profiles of patients with mood disorders show a high level of heterogeneity during the course of life (Akiskal 1989; McIntyre et al. 2022). While this variety of clinical presentation has been adequately addressed in the literature (Torre-Luque et al. 2019; Sajatovic et al. 2022; Meter et al. 2011), lithium treatment response across the lifespan is less well-studied. Specifically, it remains unclear whether the benefits of lithium use may vary in special clinical conditions. In particular, paediatric age, peripartum, and old age, should be considered separately in the pharmacological management of mood disorders, as special clinical conditions deviating from the normal distribution of patient’s characteristics. Many biological changes take place during these stages of life and may influence efficacy and safety of lithium use. During childhood and adolescence, the nervous system undergoes growth and development at a remarkable pace and may be differently influenced by lithium use. In parallel, earlier start of lithium treatment is associated with a better clinical outcome and increased probability of response to the drug (Vieta et al. 2018; Kessing et al. 2014). Similarly, the management of women with mood disorders during the peripartum period (including both pregnancy and the postpartum period, according to DSM-5) is associated with clinical concerns because of the inherent risks related to the disorders themselves as well as to their treatment (Tosato et al. 2017; Poels et al. 2018a; Yonkers et al. 2004). Regarding old age, the higher rates of physical and cognitive comorbidity in older adults, their alterations in social risk factors, and the greater likelihood of polypharmacy, all suggest that this population should be considered separately (Cooper et al. 2011). Furthermore, the age-related pharmacodynamic and pharmacokinetic changes may render older patients with mood disorders more susceptible to lithium’s adverse events (Chan et al. 2020).

Although previous reviews separately considered the use of lithium in paediatric age (Duffy et al. 2018; Amerio et al. 2018), during the peripartum period (Wesseloo et al. 2017) and in geriatric populations (Cooper et al. 2011), no study to date has synthesised the evidence on lithium efficacy and safety in these three special clinical conditions. An umbrella review can therefore overcome this shortcoming and comprehensively evaluate the benefits of lithium use across lifespan. Accordingly, the aim of this study was to evaluate and fill-out the evidence of systematic reviews and meta-analyses focusing on the efficacy and/or safety of lithium use in mood disorders occurring in the above-mentioned special clinical condition. For each special condition, we assessed the risk of bias and the degree of overlap in studies of included systematic reviews.

## Methods

### Search

We applied the *P*referred *R*eporting *I*tems for *S*ystematic Reviews and *M*eta-*A*nalyses *(PRISMA)* criteria (http://www.prismastatement.org/) to identify systematic reviews and meta-analyses reporting results on the efficacy and/or safety of lithium use in mood disorders in special life stages: (i) childhood and adolescence (patients < 18 years old); (ii) peripartum, including data on pregnancy, postpartum and lactation; (iii) old age.

Studies were still eligible when their scope was not exclusively focused on lithium (i.e. systematic reviews on the pharmacological treatment of one of the included special clinical conditions) but if they focused on mood disorders and separately reported results on lithium (i.e. meta-analytic results on lithium, synthesis tables providing results on lithium). Studies were not excluded based on their risk of bias (assessed as described below), but potential biases were highlighted and discussed in the current review. Details of the search and article eligibility criteria can be found in the supplement. Eligibility was established with consensus obtained through Delphi rounds.

### Data extraction

Specific data of the eligible full-version articles were carefully extracted and filled into the developed extraction form. The extracted outcomes, when available for each eligible study, consisted of the following: (i) number of original studies included in the systematic review; (ii) type of included studies; (iii) total number of patients treated with lithium; (iv) description of patients treated with lithium; (v) specific focus on lithium (Yes/No) (vi) primary and secondary outcomes; (vii) efficacy findings; (viii) safety findings; (ix) meta-analytic data (Yes/No), (x) conclusions.

### Risk of bias

Included systematic reviews were assessed for their risk of bias through the Risk of Bias in Systematic Reviews (ROBIS) tool (Whiting et al. 2016). There are three phases in ROBIS, including assessing relevance, identifying concerns with the review process, and judging risk of bias. Phase one of ROBIS tool includes one item, which mainly evaluates whether the participants, exposures, comparators and outcomes match the target question. The answers are “yes,” “no,” “partial,” and “uncertain”. Phase two includes four domains: (1) study eligibility criteria; (2) identification and selection of studies; (3) data collection and study appraisal; (4) synthesis and findings. The answers to phase two questions can be “yes,” “probably yes,” “probably no,” “no” and “no information”. The bias associated with each domain is judged as “low,” “high,” or “unclear” depending on the answers to each question. Phase three focuses on whether the systematic review in its entirety is at risk of bias. In this phase, the following questions are taken into account: (1) did the interpretation of findings address all the concerns identified in domains 1 to 4; (2) was the relevance of identified studies appropriately considered in review's research question; (3) did reviewers avoid emphasising results based on their statistical significance? Possible answers to these questions are the same as phase two. Based on the answers to the questions in phase three, the overall risk of bias in the systematic reviews were rated as “low,” “high,” or “unclear.” Different investigators independently evaluated the risk of bias of all the included systematic reviews, and the disagreements were resolved through consensus.

### Analysis of degree of overlap in studies

Overlap in umbrella-reviews indicates the degree to which the included reviews address the same or different primary research literature. Overlap in primary studies across systematic reviews was calculated through the Corrected Covered Area (CCA) (Hennessy and Johnson 2020). The current guidelines for generating the CCA involve first creating a citation matrix of all primary studies (rows) included for each review (columns), where primary studies, in specific reviews, are indicated with a check mark; duplicate rows (i.e., identical primary studies) are removed so that all the instances of that primary study appearing across reviews are noted in a single line. Next, calculate CCA (Pieper et al. 2014):

$$ {\text{CCA}} = \,\frac{{{\text{N}} - {\text{r}}}}{{\left( {{\text{r}} \times {\text{c}}} \right) - {\text{r}}}} $$

where N is the total number of included publications (including double counting), in evidence synthesis (this is the sum of the ticked boxes in the citation matrix); r is the number of rows (number of index publications); and c is the number of columns (number of reviews). CCA is a proportion that can be represented as a percentage.

## Results

At the end of the eligibility process, we included 20 independent trials, for a total of 8209 individuals treated with lithium. In particular, 5 systematic reviews were included in the paediatric age section for a total of 2661 individuals treated with lithium, 10 systematic reviews were included in the peripartum section for a total of 3872 individuals treated with lithium, and 5 systematic reviews were included in the old age section for a total of 1676 individuals treated with lithium.

All included studies were written in English, although this was not a prerequisite. Further information on the strategy and results of the search can be found in the Supplement. The results of our search are shown as a PRISMA flowchart in Additional file 1: Figure S1 with the reasons of exclusion.

Table 1 provides a description of the included studies, including information on study population, study design, efficacy, safety, conclusions, and limitations.

**Table 1:** Characteristics of included studies

Study	Number of primary studies	Design of primary studies	Number of individuals treated with Li^+^	Patients’ description	Specifically focused on lithium	Specific outcomes on Li^+^	Meta-analytic findings on Li^+ a^	Findings on efficacy	Findings on safety	Conclusions
Liu et al. 2011	7	Open label, double-blind studies	151	Children (< 18 years) with BD	No	Efficacy of Li^+^ for paediatric BD	No	Open-label: response rates: 23%-55%; Double-blind: 1/3 found Li^+^ to induce long-term stabilisation	–	Limited data available
Amerio et al. 2018	30	RCTs, observational studies	1320	Children and adolescents (< 18 years) with BD treated with Li^+^ monotherapy or combined with other drugs	Yes	Safety and efficacy of Li^+^ for paediatric BD	No	Li^+^ monotherapy: efficacy for acute mania in up to 50% of patients; some evidence of long-term maintenance efficacy Combination therapy: some evidence for Li^+^ + APs and Li^+^ + DVP	Li^+^ generally safe in the short term. Most common AEs: gastrointestinal, polyuria or headache	Li^+^ reasonably safe and effective in children and adolescents with BD in the short-term
Duffy et al. 2018	4	RCTs	176	Children and adolescents (< 18 years) with BD experiencing a manic or mixed episode	Yes	Efficacy and tolerability of Li^+^ for paediatric mania	Yes	Li^+^ > plc and comparable to DVP, but < Risp for treating manic/mixed episodes and comorbid ADHD	Li^+^ not associated with serious AEs, and generally well tolerated with common AEs similar to those reported in adults	Limited data to suggest that Li^+^ is effective and tolerable for some forms of paediatric mania
Pisano et al. 2019	19	Open label, RCTs	871	Children and adolescents with BD and MDD	Yes	Efficacy and safety of Li^+^ for paediatric BD and MDD	No	Li + effective for manic symptoms, both in the acute phase and as maintenance strategy. Efficacy on depressive symptoms less clear	Generally, Li + resulted relatively safe	Li + is effective and well-tolerated in paediatric BD. Further evidences are needed for other clinical indications
Yee et al. 2019	5	RCTs, open-label	143	Youths (< 18 years) with BD treated for at least 6 months	No	Efficacy and safety of Li^+^ for the maintenance of juvenile BD lasting ≥ 24 weeks	Yes	Clinical response rate: 51.1% [CI = 0 to 164]	Mean AE risk: 23.9% [CI = 18.1 to 29.7]	Li^+^ may reduce long-term morbidity in juvenile BD. Limited data
Peripartum and lactation										
Doucet et al. 2011	10	Open, retrospective, prospective, studies, case reports	73	Women with PP mood episodes with psychotic features exposed to Li^+^	No	Effectiveness of Li^+^ for the prevention or treatment of PP psychosis	No	5 studies supported the prophylactic effect of Li^+^, 3 studies supported the use of lithium in treating PP	–	Preliminary evidence suggests Li^+^ to be effective for PP psychosis prevention and treatment
Galbally et al. 2010	12	Case–control, prospective studies, case series	773	Women exposed to Li^+^ during pregnancy	No	Pregnancy AEs and child developmental outcomes (safety)	No	–	Ebstein’s anomaly, prematurity and ↑ birth weight reported. Negative results in cohort-controlled studies	Limited data available
Uguz et al. 2016	5	Case series, case reports	26	Women with BD treated with Li^+^ during lactation	No	Infant AEs (safety)	No	–	Few clinically significant AEs reported	The incidence of AEs in infants exposed to Li^+^ is very low
Pacchiarotti et al. 2016	6	Case series, case reports	35	Women with BD treated with Li^+^ during lactation	No	Infant AEs (safety)	No	–	No clinically significant AEs reported	Available data supports the use of Li^+^ as during breastfeeding
Haskey et al. 2017	2	Retrospective cohort studies	18	Women exposed to Li^+^ during pregnancy	No	Child developmental outcomes (safety)	No	–	No AEs on cognitive development	Data on Li^+^ exposure are reassuring but are both limited and low quality
Poels et al. 2018	9	Cohort studies, case reports	107	Women exposed to Li^+^ during pregnancy	Yes	Neurodevelopmental consequences for the child (safety)	No	–	Clinical cohort studies: Li^+^ associated with normal development. Case reports: Most (4/5) reported at least one AE	Interpretation is difficult due to study heterogeneity
Imaz et al. 2019	13	Case reports, case series	39	Women treated with Li^+^ during lactation	Yes	AEs or developmental outcomes in infants (safety)	No	–	80% of breasted infants showed no AE 20.% showed at least one AE	Heterogeneity and low-moderate quality of studies
Newmark et al. 2019	12	Case reports	37	Women treated with Li^+^ during lactation	Yes	Infant AEs (safety)	No	–	AEs were reported in 9.4% of breastfed infants	Limited data
Fornaro et al. 2020	29, 13 for meta-analysis	Case–control, cohort, and interventional	2,622	Women with BD treated with Li^+^ during pregnancy and the PP compared to unexposed control subjects (both women in the general population and patients with affective disorders not exposed to Li^+^)	Yes	Relapse prevention (efficacy); risk of any malformation (safety)	Yes	Li^+^ > no Li^+^ in relapse prevention (OR = 0.16, 95%CI = 0.03–0.89)	Li^+^ associated with ↑ risk of any congenital anomaly (OR = 1.81, 95%CI = 1.35 to 2.4); cardiac anomalies (OR = 1.86, 95% CI = 1.16 to 2.96); spontaneous abortion (OR = 3.77, 95% CI = 1.15 to 12.39). Compared only with unexposed mood disorder patients, significant results only for spontaneous abortion and cardiac anomalies (in 1^st^ trimester). No association with preterm birth and low birth weight	Li^+^ exposure-associated risk at any time during pregnancy is low; ↑ risk for 1^st^-trimester or higher-dosage exposure
Uguz 2020	9	Any design, including case reports and case series	142	Women with BD exposed to Li^+^ during pregnancy and the PP	No	Relapse prevention (efficacy)	No	BD recurrence rates: Pregnancy: 22.7% PP: 20.3%	–	Li^+^ effective in preventing new mood episodes in BD during the perinatal period
Advanced age										
Ross 2008	3	Prospective, RCTs	38 using Li^+^ followed by discontinuation	Patients > 65 years with MDD treated with Li^+^ augmentation to AD, followed by Li^+^ discontinuation	Yes	Relapse prevention (efficacy)	No	Recurrence rates after discontinuation: 50% relapse over ∼6 months follow-up	–	Risk of relapse in elderly patients whose Li^+^ augmentation treatment for MDD is discontinued. Limited data
Cooper et al. 2011	5	Open-label, RCTs	64	Patients > 55 years with treatment resistant MDD treated with Li^+^ augmentation	No	Response to treatment (efficacy)	Yes	The overall response rate for Li^+^ augmentation was 42% (95% CI = 21–65)	–	Replicated evidence for Li^+^ augmentation as effective in treatment resistant MDD
Rej et al. 2012	10	RCTs, case–control studies, retrospective, cross-sectional, descriptive	835	Patients > 65 years using Li^+^	Yes	Renal AEs (safety)	No	–	ARF incidence: 1.5% per person-year, CRF prevalence: 1.2% to 34%; The prevalence of NDI:1.8% to 85%	No evidence to suggest that Li^+^ should be avoided in elderly patients
De Fazio et (2017)	15	Retrospective, prospective, RCTs	701	Patients > 50 years with BD	Yes	Efficacy and safety of Li^+^ in the treatment and prevention of the mania	No	In the 2 RCTs: Li^+^ > plc and ≥ other mood stabilizers; Recent retrospective/ prospective studies: average 72% positive response to Li^+^	Less AEs at lower doses	Evidence suggests that Li^+^ is effective and well tolerated; limited evidence
Sun et al. 2018	37	Case reports	38	Patients > 65 years using Li^+^ with BD or MDD	Yes	Li^+^ toxicity in elderly (safety)	No	–	Most common AEs: neurotoxicity, renal and cardiovascular toxicity. Precipitating factors: polypharmacy, comorbidity, high Li^+^ concentration	Lower doses of Li^+^ should be used in the elderly

^a^Meta-analytic data were considered only if separately reporting results on lithium

*↑* increase(d), > superior, < inferior, ≥ superior or comparable, *∼* about, *AD* antidepressants, *ADHD* Attention-deficit hyperactivity disorder, *AE(s)* adverse event(s), *AP(s)* antipsychotic(s), *ARF* acute renal failure, *BD* Bipolar Disorder, *CRF* Chronic renal failure, *DVP* sodium divalproex, *CI* confidence interval, *Li*^+^ lithium, *MDD* major depressive disorder, *NDI* nephrogenic diabetes insipidus, *OR* odds ratio, *plc* placebo, *PP* postpartum, *RCT* randomised-control trial, *Risp* Risperidone, *SMD* standardised mean difference

### Children and adolescents

The five systematic reviews included in the children and adolescents section involved a total of 2661 individuals treated with lithium (Table 1). Among the reviews, three were specifically focused on lithium (Duffy et al. 2018; Amerio et al. 2018; Pisano et al. 2019) while the others reported results also on other pharmacological treatments for juvenile bipolar disorders (BD) (Liu et al. 2011; Yee et al. 2019). Most reviews included a small number of studies (< 10); the systematic review including the largest number of primary studies was Amerio et al. (2018), which reported data from 30 studies. One systematic review included only randomised, double blind controlled trials (Duffy et al. 2018), while the others also considered open label and observational studies (Amerio et al. 2018; Pisano et al. 2019; Liu et al. 2011; Yee et al. 2019). All reviews involved children and adolescents with BD; one meta-analysis specifically focused on children experiencing a manic or mixed episode with comorbid attention‐deficit hyperactivity disorder (ADHD) (Duffy et al. 2018). Most included reviews reported data on both efficacy and safety, while Liu et al. (Amerio et al. 2018) provided results only on efficacy. All reviews highlighted that data available were very limited.

#### Efficacy

Only Duffy et al. (2018) provided meta-analytic findings. The findings specified that there was a lack of evidence to inform the question as to the effectiveness of lithium in paediatric BD of the classical type and that most studies included prepubertal children diagnosed with protracted manic/mixed episodes mostly with comorbid ADHD. In this context, efficacy results suggest that lithium may be superior to placebo (standardized mean difference [SMD] − 0.42, 95% confidence interval [CI] − 0.88 to 0.04), it is comparable to sodium divalproex (SMD − 0.07, 95% CI − 0.31 to 0.18), but significantly less effective than risperidone (SMD 0.85, 95% CI 0.54 to 1.15). The other included reviews reported that lithium was effective for acute mania with a response rate up to 55% (Table 1). Three reviews reported some evidence of long-term maintenance efficacy. Only Pisano et al. included both patients with BD and major depressive disorder (MDD). Authors found that efficacy on depression is not clear (Pisano et al. 2019).

#### Safety

All the studies specified that lithium was generally well tolerated with common side effects similar to those reported in adults. Two studies (Amerio et al. 2018; Pisano et al. 2019) specified that most common adverse effects were gastrointestinal, polyuria or headache. (Yee et al. 2019) found that mean adverse-effect risks for lithium was 23.9%. Long term studies specifically designed to assess safety issues are lacking.

### Peripartum

The ten systematic reviews included in the peripartum section involved a total of 3872 individuals treated with lithium (Table 1). Four of the included reviews specifically focused on lithium (Imaz et al. 2019; Fornaro et al. 2020; Newmark et al. 2019; Poels et al. 2018b), while the others reported results also on other drugs used in mood disorders during the peripartum period. Most reviews included a limited number of studies (< 20); Fornaro et al. (Fornaro et al. 2020) was the larger meta-analysis and systematic review. The design of primary studies varied across reviews, including retrospective and prospective, open label, observational, and interventional studies (Table 1). Some reviews only included case reports and case series (Imaz et al. 2019; Newmark et al. 2019; Uguz and Sharma 2016; Pacchiarotti et al. 2016). Six of the included reviews focused on women with mood disorders exposed to lithium during pregnancy (Fornaro et al. 2020; Poels et al. 2018b; Doucet et al. 2011; Haskey and Galbally 2017; Galbally et al. 2010; Uguz 2020) and four during lactation (Imaz et al. 2019; Newmark et al. 2019; Uguz and Sharma 2016; Pacchiarotti et al. 2016). One study exclusively focused on postpartum psychosis (Doucet et al. 2011). Seven of the included reviews focused only on lithium safety, two focused on lithium efficacy (Table 1) and only one provided data on both efficacy and safety (Fornaro et al. 2020). The same study was also the only one providing meta-analytic findings.

#### Efficacy

Fornaro et al. (Fornaro et al. 2020) specified that lithium use during pregnancy show superior efficacy compared to non-lithium in BD relapse prevention (OR = 0.16, 95%CI = 0.03 to 0.89). The other two studies providing results on the efficacy of lithium during pregnancy were Uguz et al. (2020) and Doucet et al. (2011). The first specified that recurrence rates in women with BD using lithium during pregnancy and post-partum were 23% and 20%, respectively (Uguz 2020). The second supported the prophylactic effect of lithium in the prevention and treatment of postpartum psychosis (Doucet et al. 2011).

#### Safety

Fornaro et al. reported that lithium was associated with congenital anomalies (OR = 1.81, 95% CI = 1.35–2.41), cardiac anomalies (OR = 1.86, 95% CI = 1.16–2.96) and spontaneous abortion (OR = 3.37, 95% CI = 1.15–12.39). They specified that risk associated with lithium exposure at any time during pregnancy was low and higher for first-trimester or higher-dosage exposure (Fornaro et al. 2020). The other reviews investigating lithium safety during pregnancy were basically in line with Fornaro et al. (Fornaro et al. 2020), reporting a low absolute risk of congenital abnormalities and no adverse effects on child developmental outcomes (Table 1). Nevertheless, they underlined the dearth of available data.

The reviews focusing on lactation provided only safety results (Imaz et al. 2019; Newmark et al. 2019; Uguz and Sharma 2016; Pacchiarotti et al. 2016). The rates of adverse effects ranged between 0 and 20% (Table 1), but the reviews warned about the dearth and low quality of the primary studies.

### Old age

The five systematic reviews included in the elderly section involved a total of 1676 individuals treated with lithium (Table 1). All the included reviews exclusively focused on lithium, except one (Cooper et al. 2011). Specifically, Cooper et al. (2011) systematically reviewed studies on treatments, including lithium, for refractory depression in older people. Most reviews included a limited number of studies (< 20); only Sun et al. (2018) summarised more than 37 studies, but presented only case report data. Across the reviews, the design of primary studies varied, including retrospective and prospective, open label, and observational studies, as well as randomized controlled trials (Table 1). The included reviews presented with a high level of heterogeneity in terms of study population and specific outcomes. Two studies focused on efficacy, two on safety and only one review assessed efficacy and safety of lithium exclusively in the treatment and prevention of mania.

#### Efficacy

Two studies supported the efficacy of lithium in geriatric patients with resistant MDD (Cooper et al. 2011; Ross 2008). Nevertheless, the two reviews were substantially different with respect to study design (Table 1). Cooper et al. (2011) was the only review providing meta-analytic data for the evaluation of treatment response in resistant MDD; they specified that the overall response rate for lithium augmentation was 42%. Ross et al. (2008) aimed at quantifying the risk of relapse when lithium augmentation is discontinued in geriatric patients with MDD. Recurrence rates was 50% relapse over approximately 6 month follow-up. Considering lithium efficacy on manic symptoms, De Fazio et al (2017) found that lithium was superior to placebo and to other mood stabilizers in treating mania.

#### Safety

Three other reviews focused on lithium toxicity in patients > 65 years with mood disorders (Sun et al. 2018; Fazio et al. 2017; Rej et al. 2012).The first one focused on renal adverse events (Rej et al. 2012); the second one reviewed all the effects associated with lithium toxicity (Sun et al. 2018), the third one assessed lithium tolerability in treating mania (Fazio et al. 2017). The studies suggested that lithium may be relatively well-tolerated, but low doses should be used in the elderly. Adverse events were dose-dependent.

### Risk of bias

**Fig. 1:**
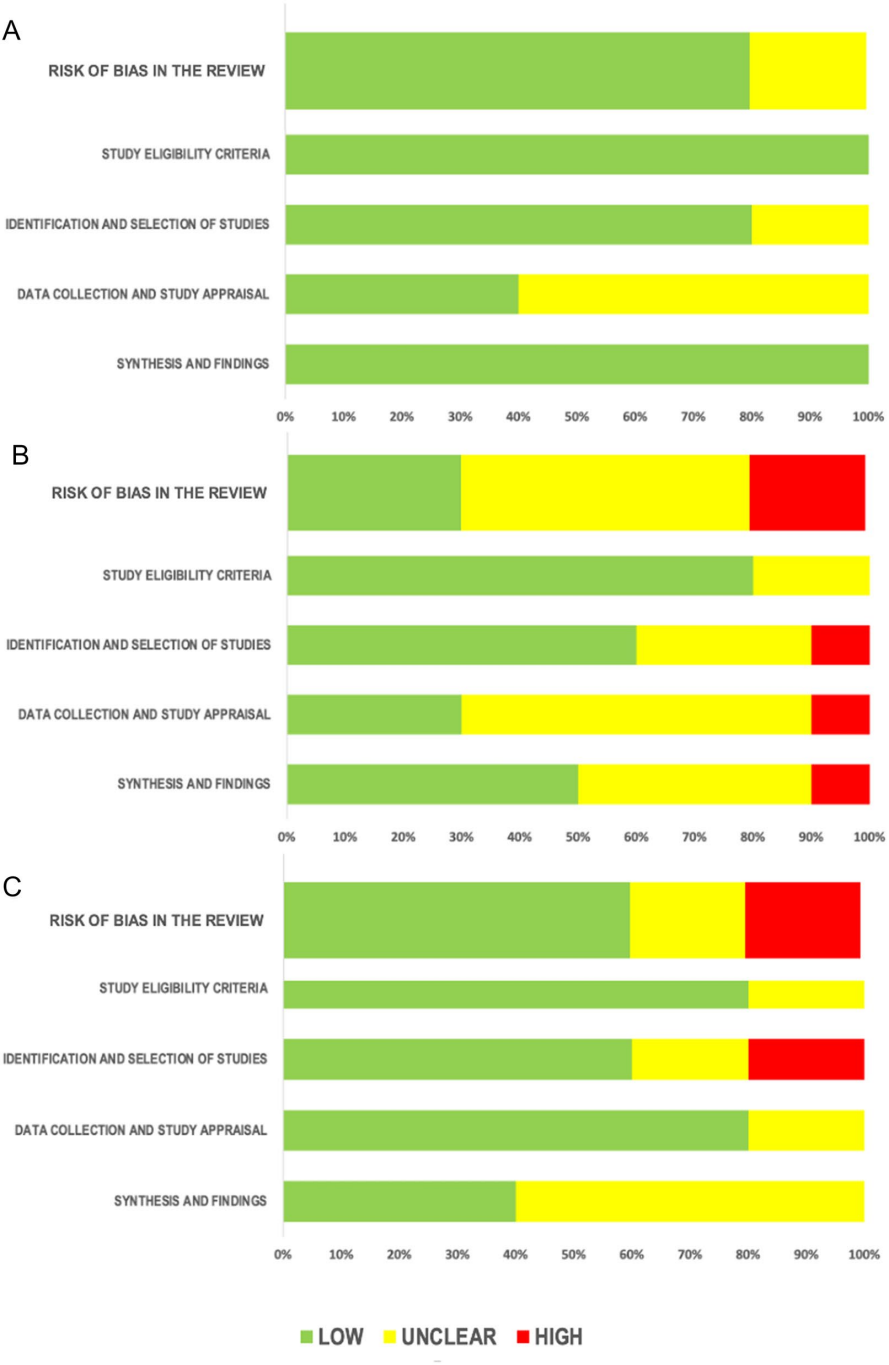
Results of risk of bias assessment. **A**, Children and adolescents; **B**, Peripartum; **C**, Old age. Phase 2 and 3 of ROBIS are depicted for all the studies included in the umbrella review. Results are presented as percentages of studies at low, unclear or high risk

The ROBIS tool was used to assess the risk of bias of the included systematic reviews. According to the results of phase 1, in all the included studies participants, exposures, comparators, and outcomes matched the target question. The results of phase 2 are shown in Fig. 1 and further detailed in Additional file 1 Results.

### Analysis of the degree of overlap in studies

Corrected covered areas (CCAs) were calculated for systematic reviews on paediatric age and peripartum. It was not possible to calculate CCAs for the old-age category, as primary studies presented with a high level of heterogeneity in terms of sample population and specific outcomes (Table 1).

Regarding the paediatric age section, the CCA for the four reviews considered was 23%. Considering in the citation matrix only randomised controlled trials, which evaluated more homogenous parameters, results in a CCA increase to 38%.

Based on the results of studies on peripartum, we decided to divide the studies into two groups, i.e., those focusing on the use of lithium during pregnancy and those focusing on lactation (Table 1). For studies on lithium use during pregnancy, the overall CCA was 10%. An additional CCA was also repeated excluding from the citation matrix case reports and case series, not consistently included in the systematic reviews on pregnancy. A CCA of 13% was obtained. All four reviews on lactation focused on safety, specifically on infant adverse events and neurodevelopmental consequences for the child after lithium exposure. The CCA for these reviews was 47%. All citation matrices can be found in the Supplement.

## Discussion

To the best of our knowledge, this is the first umbrella review assessing efficacy and safety of lithium across the lifespan by simultaneously targeting three specific life stages: childhood and adolescence, peripartum (pregnancy, postpartum and lactation), and old age.

### Children and adolescents

Regarding childhood and adolescence, the findings outline a dearth of systematic reviews on the topic. We found five systematic reviews, most of them including a small number of studies (< 10), with only one review reporting data from 30 primary studies and more than 1000 patients (Amerio et al. 2018). Nevertheless, the risk of bias for this group of studies was relatively low, with the 80% of reviews being at low risk (Fig. 1). This result corroborates the substantial agreement among the conclusions of included systematic reviews. They all supported lithium as a potential reasonably safe and effective treatment in children and adolescents (Table 1); however, they strongly underlined the limited number of available studies.

The only meta-analysis included in our review restricted this observation to prepubertal children protracted manic/mixed episodes and comorbid attention ADHD, specifying that lithium may be superior to placebo, it is comparable to sodium divalproex, and inferior to risperidone (Duffy et al. 2018). Results are not surprising and in line with robust evidences in adults, showing that antipsychotic drugs were more effective than mood stabilizers in treating mania in the short-term (Cipriani et al. 2011). Authors specifically warned about the lack of evidence to inform the question as to the effectiveness of lithium in paediatric BD of the classical type. The other included reviews reported that lithium was effective for acute mania with a response rate up to 55%. Included studies provided some evidence of long-term maintenance efficacy. Pisano et al. (2019) specified that the efficacy of lithium use in MDD is not clear. Further primary studies with larger primary sample size, as existing in the adult populations (Nunes et al. 2020), are necessary to determine lithium response rates in different mood states and in the long-term.

The included systematic reviews agreed that lithium was generally well-tolerated, with common adverse events that were similar to those experienced by adults and that usually showed a dose–response pattern. This is in line with a recent pharmacokinetic study conducted in 61 children with BD, showing that, when adjusting for body size, the pharmacokinetic parameters in paediatric patients were within the range of estimates from adults (Landersdorfer et al. 2017). Results are also in line with a large scale systematic meta-review on the adverse effects of medications in paediatric psychiatric illnesses highlighting that lithium showed the safer profile among mood stabilizers (Solmi et al. 2020).

With respect to the overlap in primary studies across systematic reviews, the CCA for the five reviews included in the paediatric group, was 23%. The main reason of this relatively small overlap could be identified in the high level of heterogeneity in the primary studies included in the systematic reviews. Heterogeneity may derive from virtual differences in different studies or be caused by various biases. Different inclusion criteria and definition may primarily cause clinical heterogeneity. Sources of heterogeneity may also derive from different study designs, specific outcomes and quality. For example, there were both randomised-control trials (RCTs) and open label studies in different systematic reviews (Table 1). Accordingly, when we considered in the citation matrix only RCTs, the CCA increased to 38%.

It is worth noticing that none of the included reviews focused on the efficacy of lithium in juvenile suicide prevention. This is an important gap to fill, given that recent meta-analytic findings, including over 2000 youths diagnosed with mood disorder, specified that the pooled incidence of suicide attempts in juvenile BD was 31.5% (Crescenzo et al. 2017). Based on the convincingly proved prophylactic activity of lithium in adulthood (Wilkinson et al. 2022), further systematic reviews and meta-analyses are required to find out whether the efficacy of lithium in suicide prevention may extend to the paediatric age as well.

### Peripartum

Regarding the peripartum period, we included a relatively large number of studies (N = 10). Based on the results, we decided to divide the systematic reviews in two groups, those focusing on lithium use during pregnancy and postpartum and those focusing on lactation (Table 1). The most comprehensive and recent meta-analysis on pregnancy and the postpartum was conducted by Fornaro and colleagues (Fornaro et al. 2020). Authors reported data on lithium efficacy and safety from over 2,000 pregnancies, comparing women treated with lithium to unexposed control subjects (both women in the general population and patients with affective disorders not exposed to lithium) (Fornaro et al. 2020). Providing meta-analytic findings, Fornaro et al. (Fornaro et al. 2020) concluded that lithium was superior to non-lithium in relapse prevention) and that the risk of any congenital anomaly associated with lithium exposure at any time during pregnancy was low. In line with recent large cohort data (Munk-Olsen et al. 2018), the risk was higher for first-trimester or higher-dosage exposures (Fornaro et al. 2020). Interestingly, the risk significantly decreased if lithium-taking patients were compared only to patients with affective disorders not taking lithium (Fornaro et al. 2020). This result highlights the importance of taking as reference adequate control groups in pregnancy studies, so to balance the benefits and risks of pharmacological intervention (Viswanathan et al. 2021; Scrandis 2017). Recent meta-analytic findings confirmed previous naturalistic observations (Rosso et al. 2016) and showed that postpartum relapse rates in BD were significantly higher among patients who were medication-free during pregnancy (66%; 95% CI = 57–75) than among those using prophylactic medication (23%; 95% CI = 14–37) (Wesseloo et al. 2016). Medication showed the same protective effect on relapse rates during pregnancy (Stevens et al. 2019). The other systematic reviews investigating lithium efficacy and safety during pregnancy were basically in line with Fornaro et al. (Poels et al. 2018a).

Three of the included reviews specifically investigated neurodevelopmental outcomes for those children exposed to lithium during pregnancy (Poels et al. 2018b; Haskey and Galbally 2017; Galbally et al. 2010). Available data were reassuring, although limited, and suggest that lithium use during pregnancy is associated with normal child neurodevelopment. This observation is in line with a very recent study founding no evidence for significantly altered neuropsychological functioning of lithium-exposed children at the age of 6–14 years (Poels et al. 2022). Specifically, authors found no association between prenatal lithium exposure and IQ and no relationship between lithium blood level during pregnancy and neuropsychological functioning (Poels et al. 2022).

Considering the studies on lactation, they were only focused on lithium safety. Authors reported lithium adverse events ranging between 0 and 20% (Table 1) during the lactation period. It should be stressed that results were based on few primary included studies, which were all case reports and case series. Future control group studies with longitudinal designs are needed to find the balance between the risk associated with lithium intake and the benefit of breastfeeding in mood disorders. This might be of particular importance, because recent data showed that there is no differences in oxytocin levels between women with depression and asymptomatic ones during observed infant feeding sessions (Whitley et al. 2020).

The overall risk of bias for the group of systematic reviews on the peripartum period was moderately high (Fig. 1). Only 30% of studies showed low risk, preventing us from being able to generalise the results from this group of studies. The main reasons are (1) several reviews failed to adopt measures to prevent the biases in the identification and selection of the primary studies; and (2) they also failed in using appropriate criteria for data collection and study appraisal and data synthesis (Additional file 1: Figure S2; Additional file 1 Results). This result may be explained by the fact the articles spanned from 2011 to 2020, a period during which the methodological standards to apply to systematic reviews changed. The same explanation could apply to the low level of overlap in primary studies across systematic reviews (10% and 13%, excluding case reports), indicating a high level of heterogeneity. A higher overlap was found for lactation studies (47%), probably depending on the small number of primary studies available on the topic (only case reports and case series) and consequently on the relatively unrestricted inclusion criteria adopted by these systematic reviews.

### Old age

Considering the geriatric population, included reviews were few and remarkably heterogeneous. Available studies supported the efficacy of lithium in geriatric patients with treatment-resistant MDD (Cooper et al. 2011; Ross 2008), or mania (Fazio et al. 2017). Remarkably, no systematic reviews summarised the efficacy of lithium in BD relapse prevention. This is an important gap that needs to be bridged, since first manic episodes rarely occur in this age group, while recurrence of BD episodes is frequent (Dunner 2017). Furthermore, none of the included reviews considered the effect of lithium on cognitive symptoms. A growing body of evidence supports the neuroprotective effects of lithium (Malhi et al. 2013). In elderly patients, in particular, a recent study showed that lithium use may influence the volume of the hippocampus (Zung et al. 2016). Starting from the first evidence obtained by Kessing and colleagues (Kessing 2004), it is also well established that lithium significantly reduces the risk to develop Dementia in elderly patients with BD (Ishii et al. 2021; Nunes et al. 2007; Velosa et al. 2020). Accordingly, a more comprehensive review of lithium efficacy on cognition may help understanding the mechanisms underlying neuroprotection in the elderly with mood disorders (Bersani et al. 2016).

Regarding safety, three of the included reviews focused on lithium toxicity and found that lithium is relatively well-tolerated in the elderly, provided that low doses are used (Sun et al. 2018; Fazio et al. 2017; Rej et al. 2012). Adverse events were dose-dependent, including all renal effects. Data are in line with previous observations in BD (Fotso Soh et al. 2019; Ljubic et al. 2021; Arnold et al. 2021) and with a 6 year follow-up study showing that median lithium serum concentration in elderly patients was 0.55 mmol/l, at the lower end of the therapeutic window of younger adults (Bocchetta et al. 2017). Another large study confirmed that higher serum lithium concentration is a risk factor for renal functioning decline in long-term lithium exposure (Tondo et al. 2017).

The overall risk of bias for systematic reviews included in the geriatric group was moderate, with 60% of studies showing a low risk (Fig. 1). It was not possible to calculate the overlap in primary studies across systematic reviews because, as shown in Table 1, articles had an extremely high level of heterogeneity in terms of efficacy and safety outcomes and patient populations. A critical point emerging from this overview is the need to establish a clear cut-off age for future systematic reviews on lithium use in the elderly. In fact, the included reviews provided different cut-offs to define the geriatric population. The adopted cut-off ranged between > 50 and > 65 years. This heterogeneity reflects the uncertainty expressed by the scientific community on this particular topic. Recently, the International Society for Bipolar Disorders Task Force proposed to consider > 50 years as a demarcation for older-age BD (Sajatovic et al. 2015). Nevertheless, it reported that many studies considered older-age BD as BD in individuals aged ≥ 60 years (Sajatovic et al. 2015).

## Limitations

Before presenting our conclusions, we must acknowledge some points that might limit the generalisability of our results. First, only published systematic reviews and meta-analyses on lithium use in paediatric age, peripartum, and old age were included, which may have omitted some important recently published individual studies. Second, the results of bias assessment showed that the included reviews, in particular peripartum and old age, had a moderately high risk of bias. This finding demonstrates that investigators should use more appropriate study eligibility criteria and data synthesis methods in future systematic reviews. Third, the number of included reviews, in particular for paediatric and old ages, was small. Future studies focused on the extremes of the Gaussian age curve are surely needed. Fourth, the relatively low degree of overlap in studies, as assessed by the CCA, requires that further systematic reviews and meta-analyses should standardise inclusion/exclusion criteria and search strategies (including an appropriate number of databases).

## Conclusions

In conclusion, this umbrella review supports the use of lithium across the lifespan, with particular reference to paediatric age, peripartum period, and old age. Lithium appears to be effective and relatively safe in these special life stages and emerges as a viable treatment option to antipsychotic drugs, already widely used (Centorrino et al. 2005). Low doses should be used in the elderly. Further studies are needed, in particular for paediatric and old ages, to confirm these initial observations. Given the high level of heterogeneity among the systematic reviews, studies with increased methodological homogeneity need to be performed from now and onwards, so that meta-analyses could obtain more sound results and inform improved patient outcomes across the lifespan.

## Supplementary Information

**Additional file 1: Figure S1.** PRISMA flow diagram of included studies. **Figure S2.** Risk of bias assessment for each systematic review. **Table S1.** CCA of the primary studies included in the systematic reviews on pediatric population. **Table S2.** CCA of all the randomized controlled trials (RCT) included in the systematic reviews on pediatric population. **Table S3.** CCA of the primary studies included in the systematic reviews on pregnancy and peripartum. **Table S4.** CCA of the primary studies included in the systematic reviews on pregnancy and peripartum without case reports

## Data Availability

All data generated or analysed during this study are included in this published article [and its Additional file 1].

## Abbreviations

ADHD: Attention-deficit hyperactivity disorder
BD: Bipolar disorders
CCA: Corrected Covered Area
MDD: Major depressive disorder
PRISMA: Preferred Reporting Items for Systematic Reviews and Meta-analyses criteria
RCTs: Randomised-control trials
ROBIS: Risk of Bias Assessment Tool for Systematic Reviews
