# Supplementary material for: Lithium use in childhood and adolescence, peripartum, and old age: an umbrella review
Source: Int J Bipolar Disord. 2023 Feb 12;11:8. doi: 10.1186/s40345-023-00287-7 (PMC9925650; doi:10.1186/s40345-023-00287-7)
Supplement: Supplementary file 1 — Additional file 1: Figure S1. PRISMA flow diagram of included studies. Figure S2. Risk of bias assessment for each systematic review. Table S1. CCA of the primary studies included in the systematic reviews on pediatric population. Table S2. CCA of all the randomized controlled trials (RCT) included in the systematic reviews on pediatric population. Table S3. CCA of the primary studies included in the systematic reviews on pregnancy and peripartum. Table S4. CCA of the primary studies included in the systematic reviews on pregnancy and peripartum without case reports [file 40345_2023_287_MOESM1_ESM.docx]

**Lithium use in childhood and adolescence, peripartum, and old age: a lifespan umbrella review**

Supplemental Material

**eMethods**

1. **Search strategy**
2. **Article eligibility**
3. **Data extraction**

**eResults**

1. **Results**
2. ***PRISMA* Flowchart**
3. **ROBIS**
4. **Study overlap**

**eMethods**

Search strategy

We conducted a systematic literature search in accordance with the *P*referred *R*eporting *I*tems for *S*ystematic Reviews and *M*eta-*A*nalyses (*PRISMA*) criteria (http://www.prisma-statement.org/) (eFigure 2) to identify systematic reviews and meta-analyses from any time to June 19 reporting results on efficacy and/or safety of lithium use in mood disorders in special life stages: (i) childhood and adolescence (patients <18 years old); (ii) peripartum, including data on pregnancy, postpartum and lactation; (iii) old age. The search keywords in PubMed (<http://www.pubmed.org>), then filtered by systematic reviews and meta-analyses, were: (i) “lithium”, “pregnancy”, “postpartum”, “peripartum”, “lactation”, “breastfeeding”; (ii) “lithium” “children”, “p(a)ediatric*”, “youth*”, “adolescen*”, “childhood”; (iii) “lithium”, “elderly”, “old age”, “geriatric”, “late life”, “late-onset” and their various combinations and permutations. The search was also extended to Web of Science (https://apps.webofknowledge.com) and Google Scholar (<https://scholar.google.de>).

The *PRISMA* flowchart from the database search and strategy used is shown in the Supplementary Results section.

Article Eligibility

We included articles that: (a) were systematic review or meta-analyses; (b) were specifically focused on one of the three special conditions considered (children and adolescents, peripartum and old age) (c) separately provided results on lithium use in one of the three special conditions considered; (d) provided results on efficacy and/or safety of lithium; (d) focused on patients with mood disorders.

Exclusion criteria were: (a) reviews that were not systematic; (b) systematic reviews focused on animal models, molecular biology, and pathophysiology; (c) systematic review or meta-analyses not providing data on efficacy and/or safety of lithium in patients with a mood disorder; (d) duplicate publications, conference articles, and systematic reviews that were still in the protocol stage.

Studies were still eligible when their scope was not exclusively focused on lithium, but if they focused on mood disorders and separately reported results on lithium. Systematic reviews and meta-analyses were included only if they synthesized findings from at least two original studies on the topic are reported results in a systematic way (i.e. meta-analytic results, tables providing synthesis of results on lithium). No language limitations were imposed.

Four authors (DJ, GSam, AF, GabS) separately inspected all titles and abstracts of articles resulting from the electronic database searches. After obtaining the full-text version of the relevant articles, the reviewers individually re-evaluated them. Eligibility was established with consensus among four authors (DJ, GSam, AF, GabS) through Delphi rounds carried out with online meetings. Three were sufficient to reach consensus (75% agreement).

**Data extraction**

Specific data of the eligible full-version articles were carefully extracted and filled into the extraction form. The extracted outcomes, when available from each eligible study, consisted of the following:

(i) number of original studies included in the systematic review; (ii) type of included studies; (iii) total number of patients treated with lithium (if not available this number was calculated from the original studies included in the reviews); (iv) description of patients treated with lithium (diagnosis and other clinical specifications) (v) specific focus on lithium (vi) primary and secondary outcomes (according to the inclusion criteria of the current review); (vii) findings on efficacy; (viii) findings on safety; (ix) meta-analytic data (meta-analytic data were considered only if separately reporting results on lithium), (x) conclusions (as reported in the original studies).

**Risk of bias**

Included systematic reviews were assess for their risk of bias through the Risk of Bias in Systematic Reviews (ROBIS) tool. Details are reported in the main manuscript. Two investigators (DJ, GSan) independently evaluated the risk of bias of all the included systematic reviews, and the disagreements were resolved through consensus.

**eResults**

The above-mentioned search produced 5644 records on PubMed, Web of Science (limiting to

pediatrics), and Google Scholar.

At end of the eligibility process, we included 20 independent trials, for a total of 8,209 patients. All the included studies were randomized controlled trials, written in English. Included studies spanned from 2008 to 2020. The results of our search are shown as a *PRISMA* flowchart in Supplementary Figure 1 with the reasons of exclusion.

**
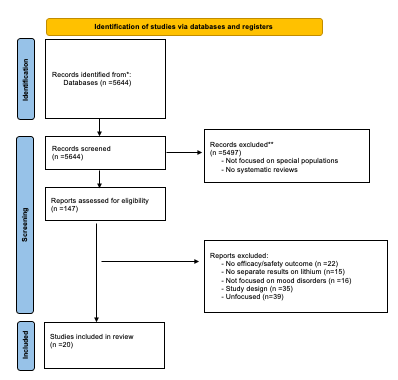
**

For more information, visit: http://www.prisma-statement.org/

**Supplementary Figure 1. PRISMA flow diagram of included studies**

**Risk of bias**

*Results of risk of bias assessment of the included systematic reviews*

The ROBIS tool was used to assess the risk of bias of the included systematic reviews.

Phase 1: According to the results of phase 1, in all the included studies participants, exposures, comparators, and outcomes matched the target question.

Phase 2: Domain one assessed the appropriateness of the study eligibility criteria. No articles were ranked with high bias due to inappropriate inclusion criteria, but three were rated as unclear because there were no pre-defined objectives or not enough information on eligibility criteria. Two of the twenty systematic reviews were rated as high risk in the second domain, which assesses the risk of bias of the methods used for study identification and selection. High risk of bias was due to imposing restrictions on the language or the date of publications, but also to an inappropriate range of sources searched. Similarly, other five reviews were rated as unclear due to lack of information about these same aspects. When evaluating the third domain on data collection and study appraisal, one systematic review was rated as high risk of bias, because insufficient efforts were made to minimize errors in data extraction. Ten systematic reviews were rated as unclear because they did not report whether the risk of bias assessment was performed and independently completed by two authors. Domain four assessed the appropriateness of data synthesis methods. One systematic review was rated as high risk of bias, due to concerns about inclusivity and the lack of sensitivity analyses, while seven were rated as unclear.

Phase 3: Overall, ten articles were rated as being at low risk, seven as having an unclear level of risk, while three articles had high risk of bias according to the results of the third phase of ROBIS (Supplementary Figure 2).

**Supplementary Figure 2**. Risk of bias assessment for each systematic review

| **RISK OF BIAS DOMAINS** | **Phase 2** | | | | **Phase 3** |
| --- | --- | --- | --- | --- | --- |
|  | STUDY ELIGIBILITY CRITERIA | IDENTIFICATION AND SELECTION OF STUDIES | DATA COLLECTION AND STUDY APPRAISAL | SYNTHESIS AND FINDINGS |  |
| **Study ID.** |  |  |  |  |  |
| **Peripartum and lactation** | | | | | |
| Galbally et al. (2010)[1] | 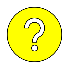 | 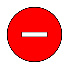 | 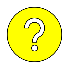 | 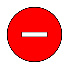 | 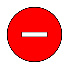 |
| Doucet et al. (2011)[2] | 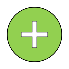 | 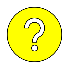 | 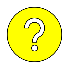 | 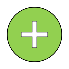 | 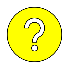 |
| Uguz et al. (2016)[3] | 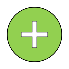 | 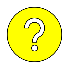 | 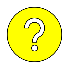 | 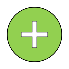 | 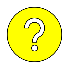 |
| Pacchiarotti et al. (2016)[4] | 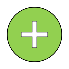 | 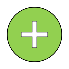 | 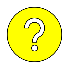 | 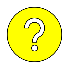 | 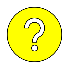 |
| Haskey et al. (2017)[5] | 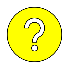 | 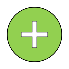 | 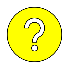 | 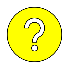 | 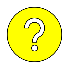 |
| Poels et al. (2018)[6] | 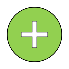 | 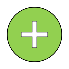 | 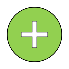 | 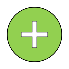 | 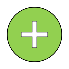 |
| Imaz et al. (2019)[7] | 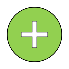 | 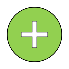 | 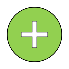 | 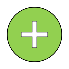 | 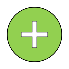 |
| Newmark et al. (2019)[8] | 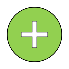 | 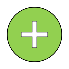 | 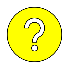 | 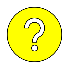 | 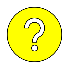 |
| Fornaro et al. (2020)[9] | 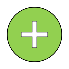 | 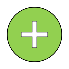 | 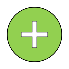 | 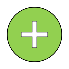 | 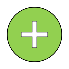 |
| Uguz et al. (2020)[10] | 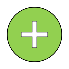 | 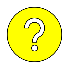 | 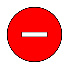 | 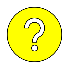 | 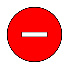 |
| **Pediatric population** | | | | | |
| Liu et al. (2011)[11] | 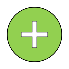 | 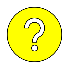 | 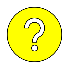 | 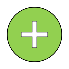 | 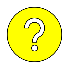 |
| Amerio et al. (2018)[12] | 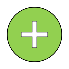 | 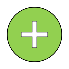 | 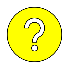 | 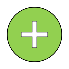 | 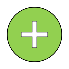 |
| Duffy et al. (2018)[13] | 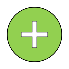 | 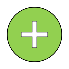 | 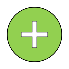 | 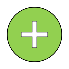 | 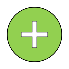 |
| Yee et al. (2019)[14] | 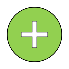 | 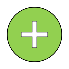 | 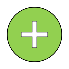 | 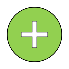 | 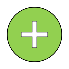 |
| Pisano et al. (2019)[15] | 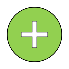 | 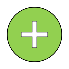 | 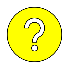 | 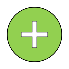 | 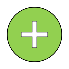 |
| **Old population** | | | | | |
| Ross et al. (2008)[16] | 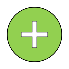 | 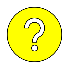 | 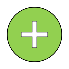 | 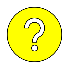 | 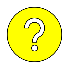 |
| Cooper et al. (2011)[17] | 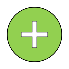 | 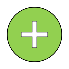 | 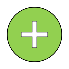 | 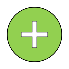 | 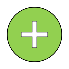 |
| Rej et al. (2012)[18] | 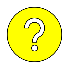 | 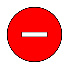 | 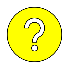 | 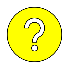 | 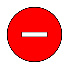 |
| De Fazio et al. (2017)[19] | 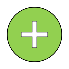 | 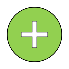 | 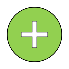 | 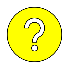 | 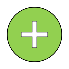 |
| Sun et al. (2018)[20] | 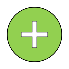 | 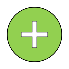 | 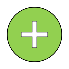 | 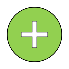 |  |
| Judgment: Low; Unclear; High | | | | | |

- 1. ***Analysis of the degree of overlap in the eligible studies***

All citation matrices addressing the degree of overlap in included studies are showed below. All the references for primary studies included in the citation matrix can be found in the original systematic reviews.

| ***eTable1:* CCA of the primary studies included in the systematic reviews on pediatric population** | | | | | | |
| --- | --- | --- | --- | --- | --- | --- |
| **Study ID.** | Liu  (2011) | Amerio (2018) | Duffy^#^  (2018) | Yee  (2019) | Pisano  (2019) | **N. of times included** |
| Kowatch et al., 2000 |  |  |  |  |  | 4 |
| Kafantaris et al., 2001 a (psycosis) |  |  |  |  |  | 2 |
| Kafantaris et al., 2003 |  |  |  |  |  | 3 |
| Pavuluri et al., 2006 a |  |  |  |  |  | 2 |
| Pavuluri et al., 2006 b |  |  |  |  |  | 1 |
| De Long et al. 1987 |  |  |  |  |  | 1 |
| Findling RL et al. 2003 |  |  |  |  |  | 1 |
| Findling RL et al. 2005 |  |  |  |  |  | 4 |
| Findling RL et al. 2006 |  |  |  |  |  | 1 |
| Findling RL et al. 2010 |  |  |  |  |  | 1 |
| Findling RL et al. 2011 |  |  |  |  |  | 2 |
| Findling RL et al. 2013 |  |  |  |  |  | 2 |
| Findling RL et al. 2015 |  |  |  |  |  | 3 |
| Geller et al, 1998a |  |  |  |  |  | 3 |
| Geller et al, 1998b |  |  |  |  |  | 2 |
| Geller 2012 |  |  |  |  |  | 3 |
| Jairam 2004 |  |  |  |  |  | 1 |
| Kafantaris et al., 1998 |  |  |  |  |  | 1 |
| Kafantaris et al., 2004 |  |  |  |  |  | 3 |
| Kowatch et al., 2003 |  |  |  |  |  | 1 |
| Landersdorfer et al. 2017 |  |  |  |  |  | 1 |
| McKnew et al. 1981 |  |  |  |  |  | 1 |
| Moore et al. 2002 |  |  |  |  |  | 1 |
| Patel et al. 2006 |  |  |  |  |  | 2 |
| Pavuluri et al., 2004 |  |  |  |  |  | 3 |
| Salpekar et al. 2015 |  |  |  |  |  | 3 |
| Strober et al. 1988 |  |  |  |  |  | 1 |
| Strober et al. 1990 |  |  |  |  |  | 3 |
| Strober et al. 1998 |  |  |  |  |  | 2 |
| Vitiello et al. 2012 |  |  |  |  |  | 2 |
| Walkup et al. 2015 |  |  |  |  |  | 2 |
| Kafantaris et al., 2001 b (mania) |  |  |  |  |  | 2 |
| Fallah et al., 2016 |  |  |  |  |  | 1 |
| Dickstein et el., 2009 |  |  |  |  |  | 1 |
| Strober et al. 1992 |  |  |  |  |  | 1 |
| **Total studies included** | 7 | 30 | 6 | 4 | 19 |  |
| Total of included studies (N)=67; Number of rows (r)=35; Number of columns (c)=5  CCA=(N-r/r*c-r)*100=**23%** ^#^ it was reported the number of manuscripts where the independent studies were descripted. | | | | | | |

| **eTable2: CCA of all the randomized controlled trials (RCT) included in the systematic reviews on pediatric population** | | | | | | |
| --- | --- | --- | --- | --- | --- | --- |
| **Study ID.** | Liu  (2011) | Amerio (2018) | Duffy^#^  (2018) | Yee  (2019) | Pisano  (2019) | **N. of times included** |
| Kowatch et al., 2000 |  |  |  |  |  | 4 |
| Findling RL et al. 2005 |  |  |  |  |  | 4 |
| Findling RL et al. 2011 |  |  |  |  |  | 2 |
| Findling RL et al. 2013 |  |  |  |  |  | 2 |
| Findling RL et al. 2015 |  |  |  |  |  | 3 |
| Geller et al, 1998a |  |  |  |  |  | 3 |
| Geller et al, 1998b |  |  |  |  |  | 2 |
| Geller 2012 |  |  |  |  |  | 3 |
| Kafantaris et al., 2004 |  |  |  |  |  | 3 |
| Pavuluri et al., 2004 |  |  |  |  |  | 3 |
| Salpekar et al. 2015 |  |  |  |  |  | 3 |
| Vitiello et al. 2012 |  |  |  |  |  | 2 |
| Walkup et al. 2015 |  |  |  |  |  | 2 |
| Kafantaris et al., 2001 b (mania) |  |  |  |  |  | 2 |
| Fallah et al., 2016 |  |  |  |  |  | 1 |
| Dickstein et el., 2009 |  |  |  |  |  | 1 |
| **Total studies included** | 4 | 14 | 6 | 2 | 14 |  |
| Total of included studies (N)=40; Number of rows (r)=16; Number of columns (c)=5  CCA=(N-r/r*c-r)*100=**38%** ^#^ it was reported the number of manuscripts where the independent studies were descripted. | | | | | | |

| **eTable3: CCA of the primary studies included in the systematic reviews on pregnancy and peripartum** | | | | | | |
| --- | --- | --- | --- | --- | --- | --- |
| **Study ID.** | Galbally  (2010) | Haskey   (2017) | Poels (2018) | Fornaro (2020) | Uguz (2020) | **N. of times included** |
| Weinstein MR (1976) |  |  |  |  |  | 2 |
| Schou M - Goldfield et al. (1973) |  |  |  |  |  | 2 |
| Jacobson SJ et al. (1992) |  |  |  |  |  | 3 |
| Kallen B. et al. (1983) |  |  |  |  |  | 2 |
| Zalzstein E. et al. (1990) |  |  |  |  |  | 2 |
| Edmonds LD et al (1990) |  |  |  |  |  | 2 |
| Schou M et al. (1976) |  |  |  |  |  | 3 |
| Austin MV (1992) |  |  |  |  |  | 1 |
| Schou M - Amdisen et al. (1973) |  |  |  |  |  | 1 |
| Weinstein MR (1975) |  |  |  |  |  | 2 |
| Kallen B. et al. (1988) |  |  |  |  |  | 1 |
| Troyer WA et al. (1993) |  |  |  |  |  | 2 |
| Grover S. et al. (2005) |  |  |  |  |  | 1 |
| Viguera AC et al. (2007) |  |  |  |  |  | 1 |
| Bogen DL et al. (2012) |  |  |  |  |  | 1 |
| Frew JR (2015) |  |  |  |  |  | 1 |
| Forsberg et al. (2012) |  |  |  |  |  | 1 |
| Van del Lugt et al. (2012) |  |  |  |  |  | 3 |
| Kozma C et al. (2005) |  |  |  |  |  | 1 |
| Morrell P et al. (1983) |  |  |  |  |  | 1 |
| Burt VK et al. (2010) |  |  |  |  |  | 1 |
| Morrell P et al. (1983) b (1 pz) |  |  |  |  |  | 1 |
| Frassetto et al. (2002) |  |  |  |  |  | 1 |
| Czeizel A et al. (1990) |  |  |  |  |  | 1 |
| Boyle B et al. (2017) |  |  |  |  |  | 1 |
| Lisi A et al. (2010) |  |  |  |  |  | 1 |
| Diav-Citrin O et al. (2014) |  |  |  |  |  | 1 |
| Newport DJ (2005) |  |  |  |  |  | 1 |
| Forsberg et al. (2018) |  |  |  |  |  | 1 |
| Patorno et al. (2017) |  |  |  |  |  | 1 |
| Frayne et al. (2018) |  |  |  |  |  | 1 |
| Munk-Olsen et al. (2018) |  |  |  |  |  | 1 |
| Bergink et al. (2012) |  |  |  |  |  | 2 |
| Rosso et al. (2016) |  |  |  |  |  | 2 |
| Viguera AC et al. (2000) |  |  |  |  |  | 1 |
| Wesseloo et al. (2017) |  |  |  |  |  | 2 |
| Restrepo et al. (2010) |  |  |  |  |  | 1 |
| Deiana et al. (2014) |  |  |  |  |  | 1 |
| Sharma et al. (2006) |  |  |  |  |  | 1 |
| Frayne et al. (2014) |  |  |  |  |  | 1 |
| **Total studies included** | 12 | 2 | 9 | 24 | 9 |  |
| Total of included studies (N)=56; Number of rows (r)=40; Number of columns (c)=5; CCA=(N-r/r*c-r)*100=**10%** | | | | | | |
| *Doucet (2011) excluded from the analysis | | | | | | |

| **eTable4: CCA of the primary studies included in the systematic reviews on pregnancy and peripartum without case reports** | | | | | | |
| --- | --- | --- | --- | --- | --- | --- |
| **Study ID.** | Galbally  (2010) | Haskey   (2017) | Poels (2018) | Fornaro (2020) | Uguz (2020) | **N. of times included** |
| Jacobson SJ et al. (1992) |  |  |  |  |  | 4 |
| Kallen B. et al. (1983) |  |  |  |  |  | 3 |
| Zalzstein E. et al. (1990) |  |  |  |  |  | 3 |
| Edmonds LD et al (1990) |  |  |  |  |  | 3 |
| Schou M et al. (1976) |  |  |  |  |  | 4 |
| Austin MV (1992) |  |  |  |  |  | 1 |
| Kallen B. et al. (1988) |  |  |  |  |  | 1 |
| Troyer WA et al. (1993) |  |  |  |  |  | 2 |
| Viguera AC et al. (2007) |  |  |  |  |  | 1 |
| Forsberg et al. (2012) |  |  |  |  |  | 1 |
| Van del Lugt et al. (2012) |  |  |  |  |  | 3 |
| Czeizel A et al. (1990) |  |  |  |  |  | 1 |
| Boyle B et al. (2017) |  |  |  |  |  | 1 |
| Lisi A et al. (2010) |  |  |  |  |  | 1 |
| Diav-Citrin O et al. (2014) |  |  |  |  |  | 1 |
| Newport DJ (2005) |  |  |  |  |  | 1 |
| Forsberg et al. (2018) |  |  |  |  |  | 1 |
| Patorno et al. (2017) |  |  |  |  |  | 1 |
| Frayne et al. (2018) |  |  |  |  |  | 1 |
| Munk-Olsen et al. (2018) |  |  |  |  |  | 1 |
| Bergink et al. (2012) |  |  |  |  |  | 2 |
| Rosso et al. (2016) |  |  |  |  |  | 2 |
| Viguera AC et al. (2000) |  |  |  |  |  | 1 |
| Wesseloo et al. (2017) |  |  |  |  |  | 2 |
| Sharma et al. (2006) |  |  |  |  |  | 1 |
| **Total studies included** | 7 | 2 | 3 | 21 | 5 |  |
| Total of included studies (N)=38; Number of rows (r)=25; Number of columns (c)=5; CCA=(N-r/r*c-r)*100=**13%** | | | | | | |
| *Doucet (2011) excluded from the analysis | | | | | | |

| **eTable5: CCA of the primary studies included in the systematic reviews on lactation** | | | | | |
| --- | --- | --- | --- | --- | --- |
| **Study ID.** | Pacchiarotti  (2016) | Uguz (2016) | Imaz  (2019) | Newmark (2019) | **N. of times included** |
| Schou M - Amdisen et al. (1973) |  |  |  |  | 3 |
| Sykes PA et al. (1976) |  |  |  |  | 3 |
| Moretti ME et al. (2003) |  |  |  |  | 4 |
| Viguera AC et al. (2007) |  |  |  |  | 4 |
| Tanaka T et al. (2008) |  |  |  |  | 4 |
| Bogen DL et al. (2012) |  |  |  |  | 4 |
| Frew JR (2015) |  |  |  |  | 3 |
| Weinstein MR and Goldfield (1969) |  |  |  |  | 2 |
| Fries H (1970) |  |  |  |  | 1 |
| Tunnessenn and Hertz (1972) |  |  |  |  | 2 |
| Skausing and Schou (1977) |  |  |  |  | 2 |
| Montgomery (1997) |  |  |  |  | 1 |
| Marin et al. (2011) |  |  |  |  | 1 |
| Shimizu et al. (1981) |  |  |  |  | 1 |
| Wise et al. (1990) |  |  |  |  | 1 |
| **Total studies included** | 6 | 5 | 13 | 12 |  |
| Total of included studies (N)=36; Number of rows (r)=15; Number of columns (c)=4  CCA=(N-r/r*c-r)*100=**47%** | | | | | |

**REFERENCES**

1. Galbally, M.; Roberts, M.; Buist, A.; Perinatal Psychotropic Review Group Mood stabilizers in pregnancy: a systematic review. *Aust. N. Z. J. Psychiatry* **2010**, *44*, 967–77, doi:10.3109/00048674.2010.506637.

2. Doucet, S.; Jones, I.; Letourneau, N.; Dennis, C.-L.; Blackmore, E.R. Interventions for the prevention and treatment of postpartum psychosis: a systematic review. *Arch. Womens. Ment. Health* **2011**, *14*, 89–98, doi:10.1007/s00737-010-0199-6.

3. Uguz, F.; Sharma, V. Mood stabilizers during breastfeeding: a systematic review of the recent literature. *Bipolar Disord.* **2016**, *18*, 325–33, doi:10.1111/bdi.12398.

4. Pacchiarotti, I.; León-Caballero, J.; Murru, A.; Verdolini, N.; Furio, M.A.; Pancheri, C.; Valentí, M.; Samalin, L.; Roigé, E.S.; González-Pinto, A.; et al. Mood stabilizers and antipsychotics during breastfeeding: Focus on bipolar disorder. *Eur. Neuropsychopharmacol.* **2016**, *26*, 1562–1578, doi:10.1016/j.euroneuro.2016.08.008.

5. Haskey, C.; Galbally, M. Mood stabilizers in pregnancy and child developmental outcomes: A systematic review. *Aust. N. Z. J. Psychiatry* **2017**, *51*, 1087–1097, doi:10.1177/0004867417726175.

6. Poels, E.M.P.; Schrijver, L.; Kamperman, A.M.; Hillegers, M.H.J.; Hoogendijk, W.J.G.; Kushner, S.A.; Roza, S.J. Long-term neurodevelopmental consequences of intrauterine exposure to lithium and antipsychotics: a systematic review and meta-analysis. *Eur. Child Adolesc. Psychiatry* **2018**, *27*, 1209–1230, doi:10.1007/s00787-018-1177-1.

7. Imaz, M.L.; Torra, M.; Soy, D.; García-Esteve, L.; Martin-Santos, R. Clinical Lactation Studies of Lithium: A Systematic Review. *Front. Pharmacol.* **2019**, *10*, doi:10.3389/fphar.2019.01005.

8. Newmark, R.L.; Bogen, D.L.; Wisner, K.Ls.; Isaac, M.; Ciolino, J.D.; Clark, C.T. Risk-Benefit assessment of infant exposure to lithium through breast milk: a systematic review of the literature. *Int. Rev. Psychiatry* **2019**, *31*, 295–304, doi:10.1080/09540261.2019.1586657.

9. Fornaro, M.; Maritan, E.; Ferranti, R.; Zaninotto, L.; Miola, A.; Anastasia, A.; Murru, A.; Solé, E.; Stubbs, B.; Carvalho, A.F.; et al. Lithium Exposure During Pregnancy and the Postpartum Period: A Systematic Review and Meta-Analysis of Safety and Efficacy Outcomes. *Am. J. Psychiatry* **2020**, *177*, 76–92, doi:10.1176/appi.ajp.2019.19030228.

10. Uguz, F. Pharmacological prevention of mood episodes in women with bipolar disorder during the perinatal period: A systematic review of current literature. *Asian J. Psychiatr.* **2020**, *52*, 102145, doi:10.1016/j.ajp.2020.102145.

11. Liu, H.Y.; Potter, M.P.; Woodworth, K.Y.; Yorks, D.M.; Petty, C.R.; Wozniak, J.R.; Faraone, S. V; Biederman, J. Pharmacologic treatments for pediatric bipolar disorder: a review and meta-analysis. *J. Am. Acad. Child Adolesc. Psychiatry* **2011**, *50*, 749–62.e39, doi:10.1016/j.jaac.2011.05.011.

12. Amerio, A.; Ossola, P.; Scagnelli, F.; Odone, A.; Allinovi, M.; Cavalli, A.; Iacopelli, J.; Tonna, M.; Marchesi, C.; Ghaemi, S.N. Safety and efficacy of lithium in children and adolescents: A systematic review in bipolar illness. *Eur. Psychiatry* **2018**, *54*, 85–97, doi:10.1016/j.eurpsy.2018.07.012.

13. Duffy, A.; Heffer, N.; Goodday, S.M.; Weir, A.; Patten, S.; Malhi, G.S.; Cipriani, A. Efficacy and tolerability of lithium for the treatment of acute mania in children with bipolar disorder: A systematic review: A report from the ISBD-IGSLi joint task force on lithium treatment. *Bipolar Disord.* **2018**, *20*, 583–593, doi:10.1111/bdi.12690.

14. Yee, C.S.; Hawken, E.R.; Baldessarini, R.J.; Vázquez, G.H. Maintenance Pharmacological Treatment of Juvenile Bipolar Disorder: Review and Meta-Analyses. *Int. J. Neuropsychopharmacol.* **2019**, *22*, 531–540, doi:10.1093/ijnp/pyz034.

15. Pisano, S.; Pozzi, M.; Catone, G.; Scrinzi, G.; Clementi, E.; Coppola, G.; Milone, A.; Bravaccio, C.; Santosh, P.; Masi, G. Putative Mechanisms of Action and Clinical Use of Lithium in Children and Adolescents: A Critical Review. *Curr. Neuropharmacol.* **2019**, *17*, 318–341, doi:10.2174/1570159X16666171219142120.

16. Ross, J. Discontinuation of lithium augmentation in geriatric patients with unipolar depression: a systematic review. *Can. J. Psychiatry.* **2008**, *53*, 117–20, doi:10.1177/070674370805300207.

17. Cooper, C.; Katona, C.; Lyketsos, K.; Blazer, D.; Brodaty, H.; Rabins, P.; de Mendonça Lima, C.A.; Livingston, G. A systematic review of treatments for refractory depression in older people. *Am. J. Psychiatry* **2011**, *168*, 681–8, doi:10.1176/appi.ajp.2011.10081165.

18. Rej, S.; Herrmann, N.; Shulman, K. The effects of lithium on renal function in older adults--a systematic review. *J. Geriatr. Psychiatry Neurol.* **2012**, *25*, 51–61, doi:10.1177/0891988712436690.

19. De Fazio, P.; Gaetano, R.; Caroleo, M.; Pavia, M.; De Sarro, G.; Fagiolini, A.; Segura-Garcia, C. Lithium in late-life mania: a systematic review. *Neuropsychiatr. Dis. Treat.* **2017**, *13*, 755–766, doi:10.2147/NDT.S126708.

20. Sun, M.; Herrmann, N.; Shulman, K.I. Lithium Toxicity in Older Adults: a Systematic Review of Case Reports. *Clin. Drug Investig.* **2018**, *38*, 201–209, doi:10.1007/s40261-017-0598-9.
